# Effects of surgically assisted rapid maxillary expansion on mandibular position: a three-dimensional study

**DOI:** 10.1186/s40510-017-0179-8

**Published:** 2017-09-04

**Authors:** Talles Fernando Medeiros Oliveira, Valfrido Antônio Pereira-Filho, Mario Francisco Real Gabrielli, Eduardo Sanches Gonçales, Ary Santos-Pinto

**Affiliations:** 1 Department of Orthodontics, School of Dentistry, São Paulo State University (UNESP), Rua Humaitá, 1680, Centro, Araraquara, São Paulo 14801-903 Brazil; 2 Department of Oral and Maxillofacial Surgery, School of Dentistry, São Paulo State University (UNESP), Araraquara, São Paulo Brazil; 3 Department of Stomatology, School of Dentistry, São Paulo University, Bauru, São Paulo Brazil

**Keywords:** Malocclusion, Palatal expansion technique, Cone beam computed tomography

## Abstract

**Background:**

This study aimed to evaluate three-dimensional changes in mandibular position after surgically assisted rapid maxillary expansion (SARME).

**Methods:**

A retrospective study was carried out with tomographic records of 30 adult patients with maxillary transverse deficiency who underwent SARME. Cone beam computed tomography scans were obtained preoperatively (T1), after expansion (T2) and 6 months after expansion (T3). Mandibular landmarks were measured with respect to axial, sagittal, and coronal planes. Repeated measures ANOVA was used for statistical analysis.

**Results:**

Clockwise rotation and lateral displacement of the mandible were observed immediately after SARME. However, mandibular displacements tended to return close to their initial values at T3.

**Conclusions:**

Clockwise rotation and lateral shift of the mandible are transient effects of SARME.

## Background

Surgically assisted rapid maxillary expansion (SARME) has been widely used to treat the maxillary transverse deficiency in adult patients [1–5]. The main effects of SARME occur transversally; however, skeletal changes in sagittal and vertical planes as a result of expansion have also been reported in the literature [1, 3, 4, 6, 7].

Despite the effectiveness of expansion in the treatment of maxillary transverse deficiencies, the possibility of causing adverse changes in patient’s profile as a result of mandibular displacement still causes concern in the indication of this procedure, mainly in hyperdivergent patients [8]. The clockwise rotation of the mandible has been reported as one of the main effects of SARME on the mandibular positioning; however, there is no consensus about the extent and stability of these changes [4, 6, 9, 10].

A possible explanation for mandibular rotation after SARME is the occlusal change due to extrusion and tipping of maxillary segments and cuspal interferences as result of expansion [9]. Previous studies that assessed changes in mandibular position after SARME have limitations since the cephalometric analysis used does not allow the three-dimensional evaluation of the mandibular positioning, consequently lateral displacement of the mandible due to expansion cannot be assessed. The use of cone beam computed tomography (CBCT) has advantages because it allows three-dimensional assessment of bilateral structures without superimposition and with minimal distortion [11–13].

This study aimed to evaluate the three-dimensional changes in mandibular positioning after SARME.

## Methods

This retrospective study assessed the CBCT records of 30 adult patients (mean age, 27.5 years; range 18.7–39.7 years; 19 females and 11 males) with maxillary transverse deficiency greater than 5 mm and unilateral or bilateral posterior crossbite. Patients with cleft lip and palate or congenital craniofacial syndromes were excluded. This study was approved by the Ethics Committee of the Araraquara School of Dentistry, UNESP, (protocol 14484713.1.0000.5416).

### Surgery and treatment protocol

Surgery was carried out under general anesthesia in hospital environment by two surgeons (V.A.P-F. and E.S.G.). SARME was performed with Subtotal LeFort I osteotomy, midpalatal suture separation, and pterygomaxillary disjunction. Patients were treated with Hyrax type appliance and activation rate of one quarter turn (0.2 mm) three times a day until the crossbite correction. The appliance activation was initiated 7 days post-operatively. After achieving the intended expansion of the maxilla width, the appliance was blocked and left in place for about 4 months. Afterward, it was removed and replaced by a transpalatal arch.

### CBCT analysis

**Fig. 1:**
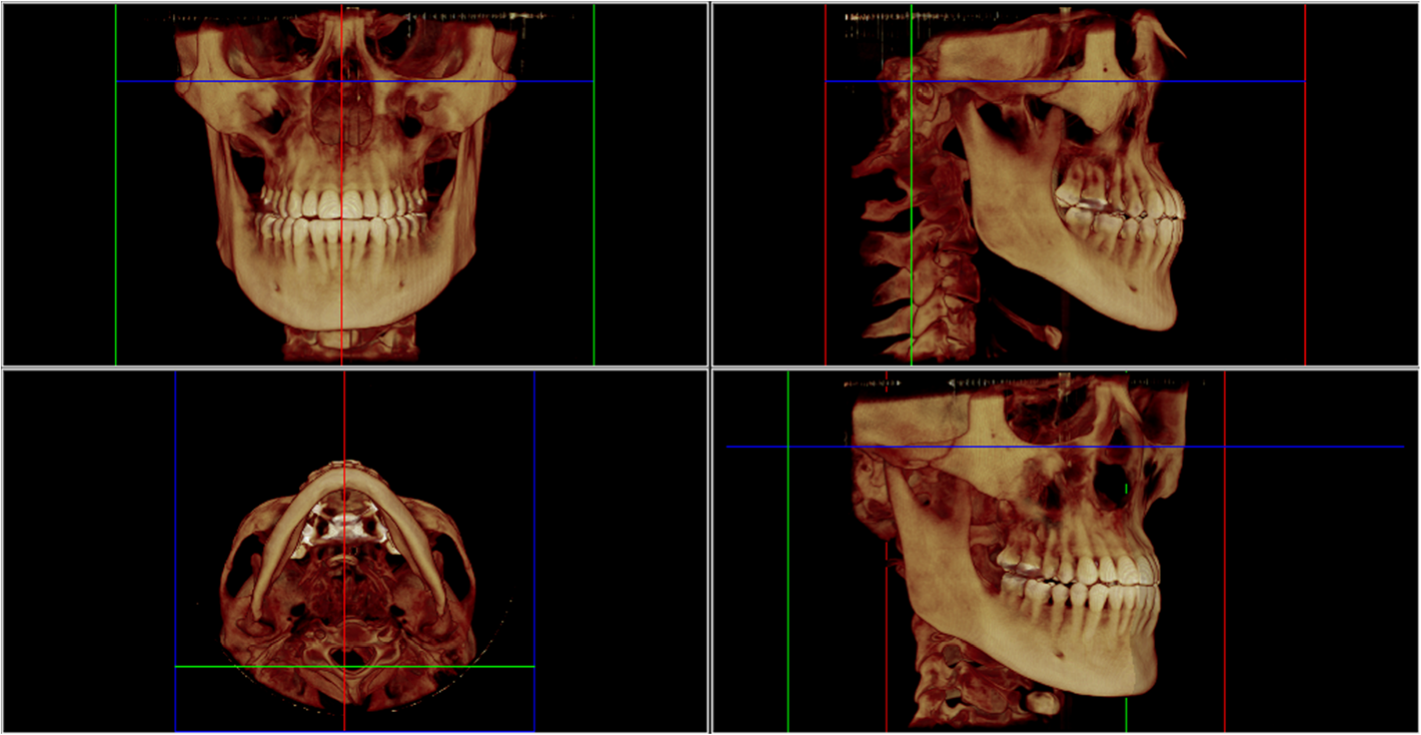
Reference planes: axial plane coincident with Frankfurt horizontal plane (*blue*), coronal plane coincident with transporionic plane (*green*), and sagittal plane coincident with midsagittal plane (*red*)

**Fig. 2:**
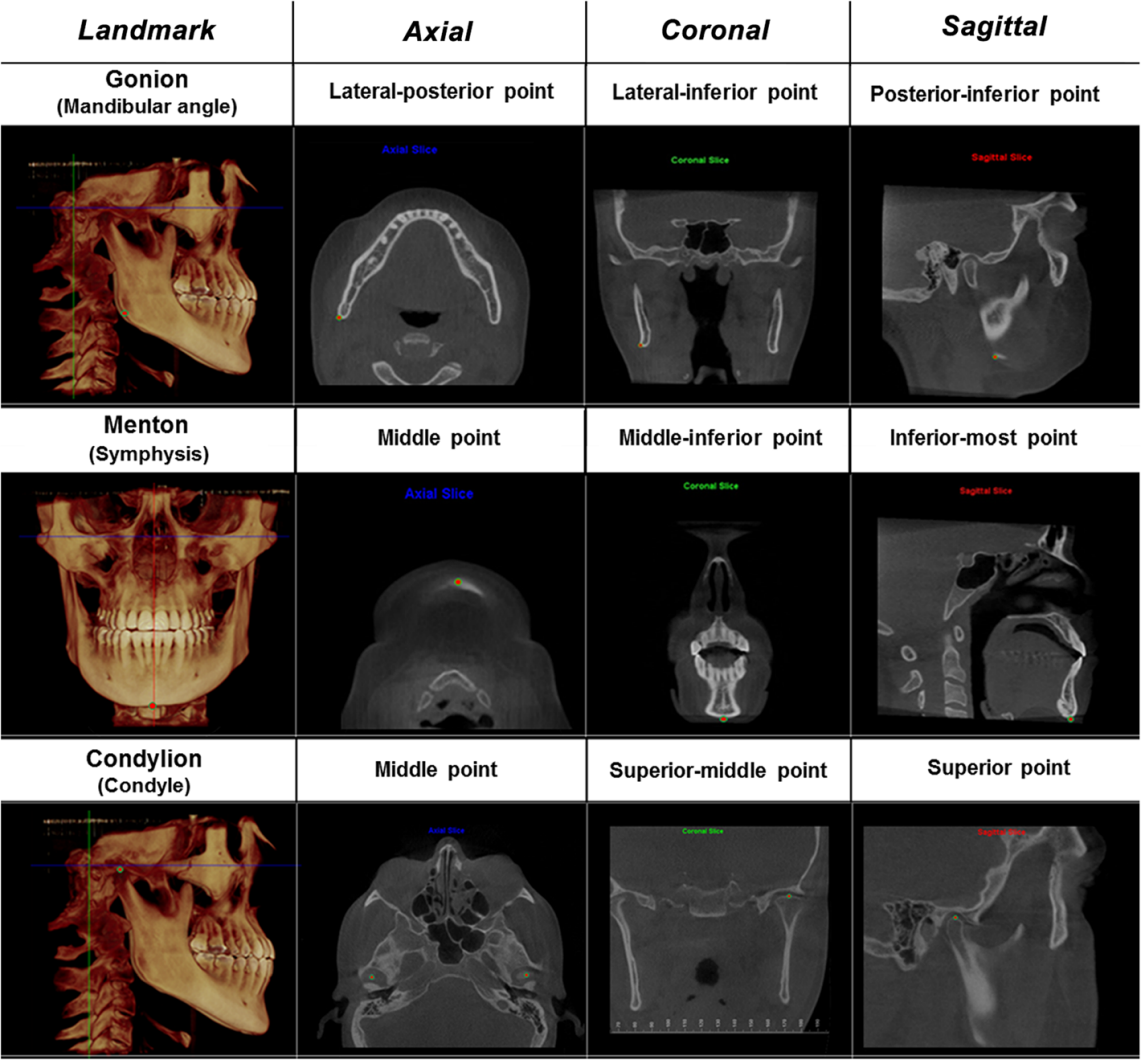
Mandibular landmarks (gonion, menton, and condylion) defined on volume rendering and multiplanar reconstruction according to axial, coronal, and sagittal slices

**Fig. 3:**
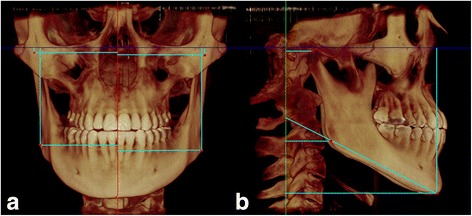
Three-dimensional representation of linear and angular measurements between mandibular landmarks and the reference planes. **a** Measurement of the mandibular landmarks related to axial plane (*blue*) and sagittal plane (*red*); **b** Measurement of the mandibular landmarks related to the coronal plane (*green*) and mandibular plane angle

CBCT scans were acquired preoperatively (T1), immediately after expansion (T2) and 6 months after expansion (T3) using an iCAT CBCT scanner (Imaging Sciences International, Hatfield, PA, USA) set up at 120 kVp, 36 mA, 0.3 mm voxel, and FOV of 17 × 23 cm. The patients were positioned sitting upright in the natural head position, and they were instructed to occlude in maximum habitual intercuspation during the CBCT scanning. The DICOM files were imported into Dolphin 3D (version 11.5, Dolphin Imaging, Chatsworth, CA, USA) for further analysis. In order to maintain the same reference planes in all time points, head orientation of each data set was standardized using orientation tool in Dolphin 3D software. The 3D orientation was performed according to three reference planes obtained from stable landmarks such as porion, orbitale, and nasion. The Frankfurt horizontal plane was defined by the right and left orbitale and the right and left porion landmarks. The transporionic plane was defined by the right and left porion landmarks, perpendicular to Frankfurt horizontal plane. The midsagittal plane was defined as the plane orthogonal to axial and coronal planes passing through nasion landmark [14]. Then, the head was moved so that the previously defined planes were coincident with the reference planes. The Frankfurt horizontal plane was oriented to match the axial plane, the transporionic plane was oriented to match the coronal plane, and the midsagittal plane was moved to match the sagittal plane (Fig. 1). Afterward, the mandibular landmarks (Menton, the right and left condylion and the right and left gonion) were defined using volume rendering and multiplanar reconstruction (Fig. 2). In order to assess the changes in mandibular position at the three time points, linear and angular measurements were performed between the mandibular landmarks and the reference planes (Fig. 3).

### Data analysis

Eighteen CBCT images were randomly chosen and assessed twice by the same calibrated examiner, with a minimum interval of 30 days. Reliability was confirmed by the intra-class correlation coefficient (ICC), which ranged from 0.929 to 0.996. The Shapiro-Wilk test was used to investigate assumptions of normality. Longitudinal changes were evaluated using repeated measures ANOVA, Greenhouse-Geisser corrections were applied for data that violated sphericity assumptions. In statistically significant results, the Bonferroni multiple comparison test was used to assess differences between time points. Data analysis were performed using SPSS 16.0 (SPSS, Chicago, IL, USA) with a significance level of 5% (*α* = 0.05).

## Results

The mandible showed a mean lateral displacement of 1.08 mm (SD = 0.93) immediately after SARME. Twenty-one patients showed lateral mandibular displacement greater than 0.5 mm after expansion. The changes in mandibular position were assessed according to the side of the mandibular displacement observed after SARME; bilateral structures were classified in contralateral or ipsilateral to the mandibular displacement observed.

Repeated measures ANOVA showed significant changes over time with respect to axial plane for menton (*p* < 0.001), and for contralateral gonion (*p* = 0.025) (Table 1). In relation to the coronal plane, only the menton measurement had significant changes (*p* < 0.001) (Table 2). However, with respect to the sagittal plane, there were changes over time for ipsilateral condylion (*p* = 0.024), contralateral condylion (*p* = 0.001), ipsilateral gonion (*p* = 0.018), and contralateral gonion (*p* = 0.029) (Table 3). Measurements of the mandibular plane angle (FMA) also changed significantly over the time of this study (*p* < 0.01) (Table 4).

**Table 1:** Mean and standard deviation of distances (millimeter) between the mandibular landmarks and the axial plane observed at the three time-point evaluations. Results for repeated measures ANOVA

Mandibular landmarks Axial plane	T1		T2		T3		*p*
	Mean	SD	Mean	SD	Mean	SD	
Ipsilateral condylion	0.79^a^	2.10	0.82^a^	1.97	0.72^a^	2.17	0.805
Contralateral condylion	0.75^a^	2.29	0.76^a^	2.00	0.87^a^	2.35	0.615
Ipsilateral gonion	58.34^a^	6.68	58.61^a^	6.79	58.47^a^	6.30	0.610
Contralateral gonion	58.49^a^	6.82	59.21^b^	6.68	59.98^a,b^	6.95	0.025
Menton	86.66^a^	8.00	88.01^b^	8.05	86.98^a^	7.92	<0.001

Different superscript letters show statistically significant differences

*T1* preoperatively, *T2* immediately after expansion, *T3* 6 months after expansion, *SD* standard deviation

**Table 2:** Mean and standard deviation of distances (millimeter) between the mandibular landmarks and the coronal plane observed at the three time-point evaluations. Results for repeated measures ANOVA

Mandibular landmarks Coronal plane	T1		T2		T3		*p*
	Mean	SD	Mean	SD	Mean	SD	
Ipsilateral condylion	15.93^a^	1.69	15.98^a^	1.70	15.76^a^	1.58	0.392
Contralateral condylion	15.91^a^	1.40	15.87^a^	1.44	15.83^a^	1.48	0.929
Ipsilateral gonion	26.09^a^	4.69	25.62^a^	5.18	26.22^a^	4.92	0.257
Contralateral gonion	25.75^a^	4.68	25.18^a^	5.70	25.92^a^	5.59	0.226
Menton	90.88^a^	8.77	89.35^b^	8.79	90.31^a^	9.13	<0.001

Different superscript letters show statistically significant differences

*T1* preoperatively, *T2* immediately after expansion, *T3* 6 months after expansion, *SD* standard deviation

**Table 3:** Mean and standard deviation of distances (millimeter) between the mandibular landmarks and the sagittal plane observed at the three time-point evaluations. Results for repeated measures ANOVA

Mandibular landmarks sagittal plane	T1		T2		T3		*p*
	Mean	SD	Mean	SD	Mean	SD	
Ipsilateral condylion	48.63^a^	3.22	49.13^b^	2.87	48.85^a,b^	2.87	0.024
Contralateral condylion	48.73^a^	3.07	47.93^b^	3.18	48.26^a,b^	46.05	0.001
Ipsilateral gonion	46.05^a^	3.05	46.67^b^	3.11	46.40^a,b^	3.05	0.018
Contralateral gonion	45.70^a^	3.81	45.17^b^	3.68	45.59^a,b^	3.58	0.029
Menton	2.23^a^	1.92	1.91^a^	1.49	2.04^a^	1.52	0.297

Different superscript letters show statistically significant differences

*T1* preoperatively, *T2* immediately after expansion, *T3* 6 months after expansion, *SD* standard deviation

**Table 4:** Mean and standard deviation of mandibular angle observed at the three time-point evaluations. Results for repeated measures ANOVA

Mandibular angle	T1		T2		T3		*p*
	Mean	SD	Mean	SD	Mean	SD	
Ipsilateral FMA	19.74^a^	4.73	20.95^b^	4.79	19.99^a^	4.60	<0.001
Contralateral FMA	19.57^a^	4.55	20.30^b^	4.76	19.67^a^	4.80	0.003

Different superscript letters show statistically significant differences

*FMA* mandibular plane angle, *T1* preoperatively, *T2* immediately after expansion, *T3* 6 months after expansion, SD standard deviation

Multiple comparison test revealed differences in the menton measures between T1 and T2 with respect to the axial plane (1.35 mm) and to the coronal plane (−1.53 mm), showing downward and backward movement of this landmark immediately after SARME (Tables 1 and 2). However, the assessment at T3 revealed a relapse of these movements (T3-T2, *p* < 0.05). Similar changes were found for measures of mandibular plane angle (FMA), indicating a transitional clockwise rotation of the mandible after expansion.

Changes in mandibular landmark measures with respect to sagittal plane confirm the lateral movement of the mandible immediately after SARME (T2-T1, *p* < 0.05); however, no significant change was observed between T2 and T3 neither between T1 and T3 (Table 3).

## Discussion

The possibility of causing adverse changes in patient’s profile as a result of mandibular displacement still causes concern in indicating maxillary expansions [8]. Clockwise rotation of the mandible with an increase in lower facial height has been reported as a side effect of SARME [6, 9]. In fact, our study found a clockwise rotation of the mandible immediately after SARME. This movement was represented by an increase in the values of the FMA as well as downward and backward displacements of the menton.

However, according to our results, the mandibular rotation seems to be a transient movement as the values observed 6 months after SARME (T3) tended to return close to their initial values (T1). Altug-Atac et al. [6] and Gunbay et al. [9] reported clockwise rotation of the mandible after SARME whereas Parhiz et al. [4] and Iodice et al. [10] did not observe significant rotational movement of the mandible. Methodological differences among these studies and assessment in different time points justify the divergence in their findings on mandibular rotation. The first authors carried out the assessment after a short period following SARME whereas the other authors conducted a later evaluation. Our findings agree with the studies found in the literature since a transient increase in the mandibular plane angle was observed.

Our findings showed that besides the clockwise rotation, previously reported in literature, there is also a lateral displacement of the mandible immediately after SARME. However, it was not related with the type of crossbite presented previously. Variations on mandibular displacement could be observed among the patients, even in those with unilateral posterior crossbite. The lack of a pattern for mandibular displacement can be explained by individual changes in the pattern of occlusion following the expansion, such as in asymmetric expansion [15]. Thus, the direction to which the mandible will move after SARME becomes unpredictable in adult patients, in contrast to the correction of postural asymmetry found in children with functional unilateral posterior crossbites [16].

Changes observed in condylion and gonion landmarks with respect to the sagittal plane occurred because the analysis was performed considering the mandibular displacement. So, one would expect an increase in the distance from the landmarks ipsilateral to mandibular displacement to the midsagittal plane, as well as a decrease in the distance from the contralateral structures to the same plane. Thus, even though an average displacement of 1.08 mm had been observed in menton between T1 and T2, it was not possible to predict the direction of this change since this landmark can move away or closer to the midsagittal plane as a result of the mandibular movement after SARME. Despite this changes occur at T2, there was a tendency to return to original position 6 months after expansion, so that no significant difference was observed between T3 and T1. Additionally, mandibular lateral movements were small and showed no clinical relevance.

Mandibular movements take place in three dimensions; thereby, bilateral mandibular structures may show distinct behaviors during SARME. Such fact was observed in vertical changes of the gonion, which was significant only to the contralateral side to the mandibular displacement. This resulted in different values of the mandibular plane angle between the ipsilateral and contralateral sides, although both have shown a significant increase.

## Conclusions

This study suggests the presence of mandibular displacement in most patients after SARME; however, the direction of this displacement cannot be predicted. Clockwise rotation and mandibular lateral displacement are transient effects of SARME.

## Abbreviations

CBCT: Cone beam computed tomography
SARME: Surgically assisted rapid maxillary expansion
